# Influence of Ovocystatin on Aβ42 soluble oligomeric and fibril formation in in vitro studies

**DOI:** 10.1192/j.eurpsy.2024.781

**Published:** 2024-08-27

**Authors:** B. Stańczykiewicz, M. Piksa, T. Goszczyński, K. Gołąb, B. Konopska, A. Zabłocka

**Affiliations:** ^1^Department of Psychiatry, Wroclaw Medical University; ^2^Department of Microbiology; ^3^Department of Experimental Oncology, Hirszfeld Institute of Immunology and Experimental Therapy, Polish Academy of Sciences; ^4^Department of Pharmaceutical Biochemistry, Wroclaw Medical University, Wroclaw, Poland

## Abstract

**Introduction:**

Alzheimer’s disease is characterized by the presence of β-amyloid deposits in senile plaques and brain vessels. β-amyloid stimulates the glial release of proinflammatory cytokines, reactive oxygen species (ROS), or nitric oxide (NO), which are potentially toxic to neurons. One potential therapy for Alzheimer’s disease is the use of agents that inhibit the aggregation and formation of insoluble β-amyloid deposits in the brain, or break down the aggregates that have already formed, thus preventing their toxicity.

**Objectives:**

This study aimed to evaluate the effect of ovocystatin on the formation and destabilization of β-amyloid aggregation.

**Methods:**

The effect of ovocystatin on β-amyloid aggregation was determined by Thioflavin T (ThT) Assay and Transmission Electron Microscopy (TEM). The impact on PC12 cell viability was determined by MTT assay.

**Results:**

Ovocystatin can interact directly with Aβ_42_, inhibiting its aggregation and reducing the toxicity induced by aggregated forms of β-amyloid. All effects are dose-dependent. Additionally, a significant increase in the PC12 cell viability treated simultaneously with Aβ_42_ and ovocystatin was observed.

**Conclusions:**

Ovocystatin may be an important factor in the prevention and treatment of Alzheimer’s disease by regulating the conversion of monomeric β-amyloid into larger and potentially more toxic particles. However, the mechanisms of inhibition of amyloid fibrillar protein formation and/or destabilization by ovocystatin are still unclear and require further investigation.

**Disclosure of Interest:**

None Declared

